# AI solutions for evolutionary genomics of nonmodel species

**DOI:** 10.1093/evlett/qrag004

**Published:** 2026-03-03

**Authors:** Michael DeGiorgio, Sandipan Paul Arnab, Matteo Fumagalli

**Affiliations:** Department of Electrical Engineering and Computer Science, Florida Atlantic University, Boca Raton, FL, United States; Department of Biomedical Engineering, Florida Atlantic University, Boca Raton, FL, United States; Center for Omics technologies and Data Engineering, Florida Atlantic University, Boca Raton, FL, United States; Department of Electrical Engineering and Computer Science, Florida Atlantic University, Boca Raton, FL, United States; Center for Omics technologies and Data Engineering, Florida Atlantic University, Boca Raton, FL, United States; School of Biological and Behavioural Sciences, Queen Mary University of London, London, United Kingdom; The Alan Turing Institute, London, United Kingdom

**Keywords:** evolutionary genomics, adaptation, population genetics, positive selection

## Abstract

As large-scale genomic datasets are becoming abundant, new questions can now be posed in evolutionary biology. Although innovative methodological approaches are constantly developed to test these new hypotheses, their application to the study of nonmodel species is hampered by technical challenges associated with such systems. In recent years, artificial intelligence (AI) solutions, mostly in the form of deep neural networks, have been successfully introduced to analyse genomic data from nonmodel species. Here, we highlight the latest trends in deep learning to infer demographic history and signals of natural selection, and offer novel research directions to develop AI algorithms for the study of nonmodel organisms. Specifically, we identify strategies to process data missingness and uncertainty, to infer selective events in the face of unknown genomic and demographic parameters, and to generate interpretable and explainable predictions. We demonstrate our arguments by showcasing an original implementation to detect selective sweeps from an experimental setting with low sample size, uncertain sequencing data, and unknown demographic model, as typical in studies of nonmodel species. We argue that the study of nonmodel organisms is an opportunity to develop general-purpose data-driven methodologies for evolutionary inferences. Fair sharing of resources and inclusive frameworks are key to enabling researchers to benefit the most from this new wave of technologies.

## The rise of AI in evolutionary studies

The inference of evolutionary events and processes from genomic data have been profoundly transformed in the last decades. With large-scale genomic datasets becoming available, novel questions can now be posed and innovative methodological approaches must be developed to test these new hypotheses. Traditionally, technological advancement has focused on improving our understanding of the evolution of model species from high-quality genomic data. Although this framework allowed robust stepwise methodological progress, studies that attempted to apply state-of-the-art inferential tools to nonmodel species have faced tremendous challenges.

Here, we define nonmodel species as organisms that are not simple and tractable by design and were not originally used to study a broad question in biological sciences. As a consequence, protocols and computational tools applicable to these systems have not been developed at the same pace as in the study of model organisms. However, nonmodel organisms provide new avenues to discover fundamentals in biology by exploiting their uniqueness (Russell et al., 2017). High-throughput sequencing technologies have been instrumental in paving the way for a reevaluation of the study of nonmodel species from a population genomics perspective (Ellegren, 2014).

Nonmodel species often exhibit biological and genomic features that violate assumptions from even the most recently proposed evolutionary models. Additionally, sequencing experiments on nonmodel species tend to generate lower-quality and sparser data than their model species counterparts. Therefore, evolutionary inferences for nonmodel species rely on old and ineffective methodologies or on *ad hoc* changes to the latest methods, the latter approach often leading to suboptimal performance.

During the last five years, an alternative cutting-edge strategy for evolutionary inferences has emerged. Using our ability to efficiently generate synthetic training data, deep learning algorithms, a branch of artificial intelligence (AI) technologies, have been rapidly adopted by the community due to their flexibility and high performance (Huang et al., 2024). Although initially tested on standard datasets, some of these software have the potential to analyse data from nonmodel species and perform tasks previously deemed inaccessible using classic statistical modelling (Korfmann et al., 2023; Smith et al., 2023).

Given the potential of deep learning for inferring demographic history and signals of natural selection in nonmodel genomes, our goal is to provide a roadmap and discussion of the potential pitfalls. We will cover how deep learning can be applicable to experimental data suffering from technical and conceptual challenges typically associated with the investigation of nonmodel organisms. We also present an original implementation (see Supplementary Material for details) to illustrate the potential of AI to tackle a challenging task in the field: the robust detection of selective sweeps in the face of data and demographic uncertainty. We propose new solutions for data representation, training, and explainability that are particularly suitable in these circumstances. Although this contribution focusses on population genomic applications for neutral and adaptive inferences, deep learning is now widely used in phylogenetics and phylogeography (Mo et al., 2024; Nesterenko et al., 2025; Perez & Gascuel, 2025; Smith & Hahn, 2023). We argue that some of our considerations can be directly transferred to these different domains.

## Limited and uncertain genomic data

**Figure 1:**
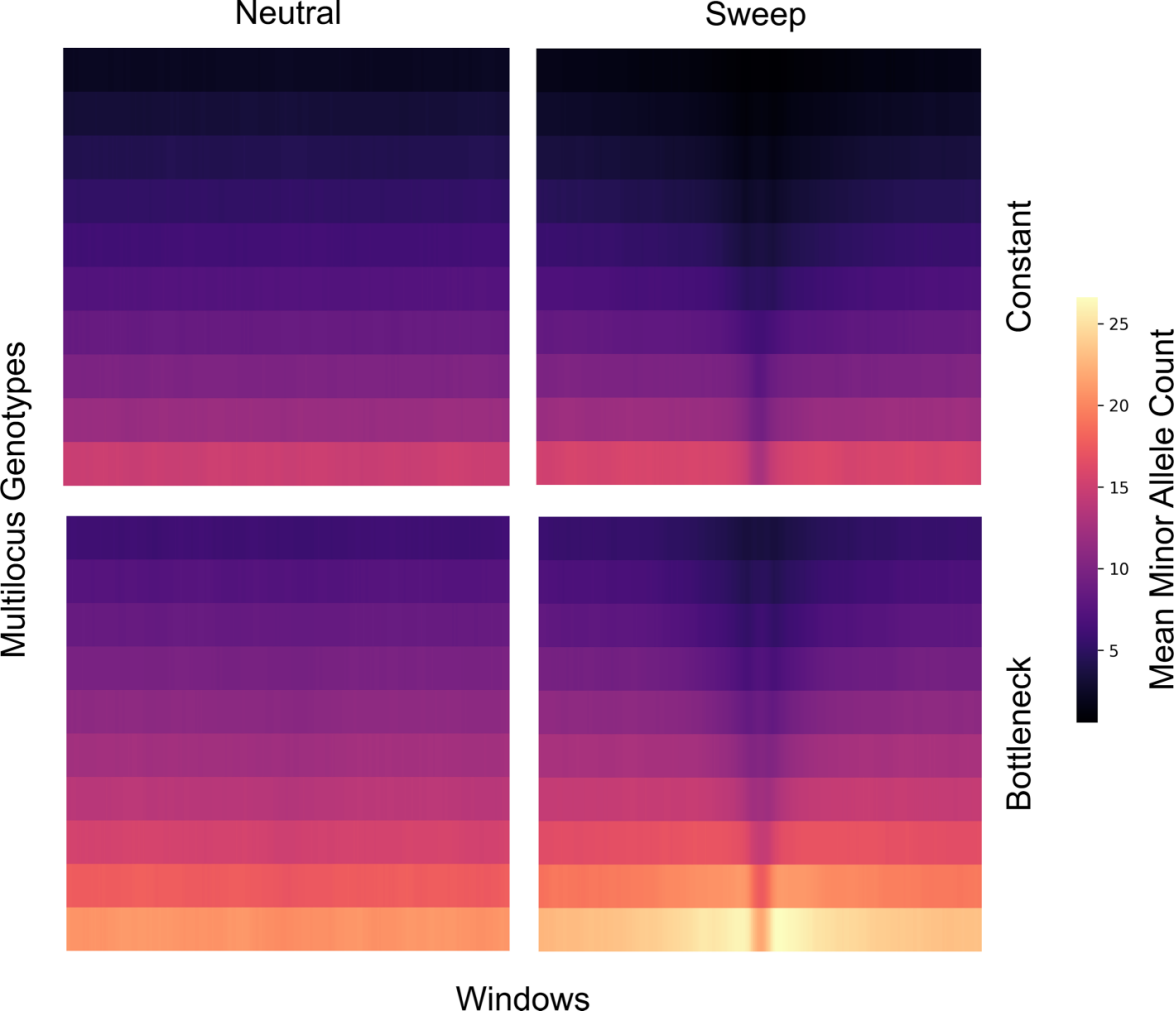
Heatmaps summarizing mean multilocus genotype variation across genomic windows for 10 sampled diploid individuals from simulated neutral and selective sweep (positive selection) settings. Darker colours represent genotypes with more major alleles than brighter colours. Selective sweeps have a tall dark segment within the central image columns (where simulated beneficial mutations arise), reflecting an extended region of low haplotype diversity due to high frequency major alleles driven by selection. Images are generated using the framework of (Arnab et al., 2025b), with the constant model (top row) used for model training and the bottleneck model (bottom row) used for model testing. Specifically, the bottleneck model has considerable genotype calling uncertainty: sequencing reads were used to capture variation at a read depth of 5x with an error rate of 0.005, and then expected minor allele counts for each genotype were obtained from estimated genotype likelihoods through ANGSD (Korneliussen et al., 2014). Due to demographic differences and genotype calling uncertainty in the bottleneck model, this situation represents a case of domain shift (Figure 2).

Despite early attempts to use supervised machine learning and neural networks for population genetic inferences (Schrider & Kern, 2018; Sheehan & Song, 2016), the disruptive nature of AI in the field emerged when researchers developed algorithms that received genome alignments as input (Chan et al., 2018; Flagel et al., 2019; Torada et al., 2019) (see Figure 1), in contrast to summary statistics. These algorithms relied on the application of convolutional neural networks (CNNs), an architecture used primarily in computer vision (Krizhevsky et al., 2012), to sequence alignments. By not compressing data into a finite set of summary statistics, these algorithms provide better performance in common population genetic tasks than competing simulation-based approaches using summary statistics (Isildak et al., 2021; Sanchez et al., 2021). However, manipulation of sequencing alignments could effectively mimic the information in classic summary statistics (Cecil & Sugden, 2023). This framework of working directly with genome alignments typically implies that high-coverage whole-genome sequencing data can be obtained from a sufficiently large sample of individuals, and a reference panel is available for imputation and phasing. As such, most studies employ deep learning algorithms on high-quality population genomic data of model species, such as humans (Villanea & Schraiber, 2019) and fruit flies (Ray et al., 2024), or organisms of biomedical interest, such as disease vectors (Eneli et al., 2025; Ketchum et al., 2025; Xue et al., 2021). The studies were able to confirm or reveal novel signatures of natural selection or demographic changes.

Genomic data for nonmodel species often exhibit technical issues as a result of challenges associated with the experimental design. For instance, due to constrained funding or sampling opportunities, sample sizes may be limited and sequencing depth and coverage could be shallow. This setup leads to uncertain genotype calls and high missingness rates (Nielsen et al., 2011), preventing any meaningful imputation and phasing if a reference panel is not available (Figure 1, bottom row). This issue is particularly relevant for endangered species whose specimens are limited to museum collections (Bi et al., 2013). Under these conditions, biases are introduced by PCR amplification to circumvent the expected low DNA yield and general poor sample quality. In some cases, whole genome sequencing is not a viable option, and the use of alternative cost-effective sequencing strategies (e.g., pooled or restriction site-associated DNA sequencing) will increase data sparsity and hamper any attempts to reconstruct genotypes and haplotypes. In fact, obtaining individual genomic data is a challenge for species with large genomes, such as vascular plants, or with small body mass, such as several marine invertebrates. The absence of data from a reference genome and a putative outgroup species generates poor mappability (especially in highly repetitive genomes) and limits any polarisation of alleles, diminishing the power to detect evolutionary processes.

In addition to technological challenges, several biological and ecological characteristics of nonmodel species are incompatible with most of the existing AI solutions for population genomic data analysis. In fact, we often lack prior knowledge of the genetic structure, taxonomic boundaries, polyploidy levels, sexual versus asexual reproduction status, and mutation and recombination rates, among many other factors. All these characteristics may exclude the study of nonmodel species to benefit from the recent wave of methodological advances in inferring and using ancestral recombination graphs (ARGs) for population genetic inferences (Nielsen et al., 2025), with few notable exceptions (Ishigohoka & Liedvogel, 2025; Korfmann et al., 2024; Pieszko et al., 2025).

One of the main issues with genomic data is that high rates of missingness do not occur randomly and therefore standard tools may not be applicable (Pigott, 2001). This consideration is crucial, as model efficiency will largely depend on our ability to impute or deal with missing data. Missingness patterns can be associated with local genomic features such as GC content, homology, repeat density, which may be difficult or impossible to model in advance. Missingness can also occur when merging datasets or samples sequenced under different experimental conditions (e.g., batch effect). If we assume a representation of population genomic data as alignment of genotypes across samples for one or multiple populations or (sub)-species, then sequencing genomic data is a case of a “missing not at random” scenario (Rubin, 1976): missing data in one entry (e.g., one genotype for a particular sample) are dependent on missingness in other entries (e.g., genotypes in linked sites and/or in samples sequenced in the same batch). Under these conditions, missing patterns are roughly monotones, as dependence is restricted to nearby sites and samples. However, the structure of the data (e.g., linkage disequilibrium due to physical proximity) is desired to be maintained. Additionally, high-dimensionality of data (e.g., temporal sampling), dependency on unobserved or unknown characteristics (e.g., contamination), and data statistical uncertainty (e.g., encoded in genotype likelihoods and posterior probabilities) contribute to a rich and complex missingness pattern that could be challenging to tackle. For example, removing blocks of missing or uncertain genotypes would bias global estimates of genetic diversity or differentiation if missingness is unevenly distributed across populations. More worryingly, the impact of missigness on downstream inferences is yet to be fully investigated, especially for supervised learning tasks. As an illustration, imputing genomic data for museum specimens using contemporary samples would dilute any natural selection signals that originated in more recent times (because historical samples would appear genetically closer to modern ones (Poyraz et al., 2024)).

Although missingness should be minimised when collecting data, it is often not possible when sampling nonmodel organisms. The integration of other omics datasets, such as transcriptomics or epigenomics, may alleviate data sparsity on the one hand but further increase the non-randomness of missing data on the other. Therefore, this scenario of “structured missingness” (Mitra et al., 2023) could potentially prevent the application of AI to genomic data from nonmodel species if not taken into account properly. The literature is rich in technical solutions to ameliorate the effects of missing or uncertain data in evolutionary genomics. In general, automated data filtering pipeline or visual exploration and manual correction of missingness patterns are typically performed, often paired with *post hoc* analyses on the effect of data filtering and imputation. In the context of AI technologies, strategies to correct for low-quality data include the calculation of expected genotype counts in windows (Gower et al., 2021) (Figure 1, bottom row), the use of masks and calibrations, and the *a priori* assessment of missingness of model performance through simulations (Perez et al., 2025). It is worth mentioning that AI has been successfully deployed for genotype imputation (Naito et al., 2021) and some supervised machine learning methods to detect selection can deal with a certain proportion of missing data (Sugden et al., 2018).

One potential solution to using existing AI solutions for evolutionary inferences from uncertain genomic data is to rely on the calculation of informative summary statistics. Probabilistic approaches allow the unbiased estimation of genetic diversity and differentiation metrics from sparse and low quality data (Korneliussen et al., 2014; Lou et al., 2021). These summary statistics can then be used as input features for AI algorithms (Lauterbur et al., 2023b). One significant drawback of this approach is that the synthetic training data will not explicitly incorporate missing or unknown data. Parametrisation of the model and simulation of sequencing experiments can be included (Altinkaya et al., 2025), but at the cost of dramatically increasing the complexity and computational time. It is also important that the algorithm does not learn from the missingness patterns if the latter is associated with spurious features. Furthermore, by design, information is lost due to genome compression to a limited number of summary statistics. Alternatively, providing spatial distributions of allele frequency as features could represent a trade-off between preserving information and providing unbiased summary statistics (Arnab et al., 2025a; Whitehouse & Schrider, 2023).

A more interesting line of research could involve exploiting structured missingness to understand its patterns and potentially learn informative features. Networks are well-studied mathematical objects that embed the topology of the data. Therefore, missingness patterns in two or more genetic loci or sequenced samples can be described, for instance, by simplicial complexes, triangulated topological spaces, which are used to efficiently compute topological invariants of the data (Torres & Bianconi, 2020). In biological applications, simplicial complexes have been used for clustering from gene expression analysis (Govek et al., 2019). One advantage of this data representation is that it is amenable to further geometrical and statistical analyses. One of them, persistent homology, calculates how simplicial complexes are maintained by varying a distance threshold and recording their number in barcodes. Persistent homology allows for the discovery of persistent patterns in the topological representation of data, with such patterns representing genuine features rather than noise (Carlsson, 2020). Intriguingly, the space of persistent barcodes embeds features (e.g., birth and death of bars), which are suitable for subsequent AI analysis. Therefore, the analysis of missingness patterns can be a direct springboard to novel inferences. Characterising missigness patterns using topological data analysis could also provide a new framework to determine when imputation or data filtering is appropriate and, when not, provide the foundation on which to build new tools to do so appropriately.

Data missingness in nonmodel species represents an opportunity to apply structured missingness models and topological data analysis to population genomic data, an approach that will naturally favour applications to model systems as well. Therefore, the challenges associated with genomic data from nonmodel species can be mitigated by appropriate preprocessing steps or by choosing a suitable algorithm that accommodates the specific features of the data under investigation.

## Rich feature generation and handling genetic and demographic uncertainty

A major complication with applying AI to evolutionary studies in nonmodel species is the uncertainty associated with the genetic and demographic parameters of the organisms of interest, as well as the adaptive processes that may be shaping their genomic landscapes. These factors are crucial, as most AI models for evolutionary and population genetic inference depend on synthetic training data produced from simulations that must encode assumptions about the processes generating genomic variation. We consider these various inferential hurdles, beginning with the latter scenario. Classical summary-statistic and likelihood-based approaches, as well as early machine- and deep-learning methods for detecting adaptive footprints, rely on specifying the expected patterns of variation produced by a given process. For example, when detecting positive selection, one might expect genomic regions characterised by long haplotypes with reduced variation, together with an allele frequency spectrum skewed towards rare alleles (Vitti et al., 2013). However, positive selection can take many forms, with some producing patterns that are more nuanced, such as adaptive introgression (Racimo et al., 2016; Setter et al., 2020) or staggered sweeps (Assaf et al., 2015). In such cases, approaches that allow models to learn features directly from the data become particularly valuable.

One common approach is to learn feature representations from image encodings of genome alignments (Figure 1), which provides a flexible way to capture a broader spectrum of adaptive signals without requiring predefined expectations. CNNs have proven to be particularly effective in extracting rich features from these inputs for downstream predictive models (Chan et al., 2018; Flagel et al., 2019; Isildak et al., 2021; Torada et al., 2019). These models capture local features within images in their initial layers and higher-order features in deeper layers. Such models provide increasingly abstract representations that facilitate downstream tasks, such as distinguishing patterns of natural selection from neutrality. However, they typically contain an enormous number of parameters, which requires substantial computational resources, energy, and data to optimise effectively. This consideration is especially problematic for data from nonmodel organisms, which are often noisy or sparse, yet may require more expressive models to capture meaningful patterns. As a solution, a recent study employed deep CNNs as feature extractors that had been pre-trained on massive image databases unrelated to genetics or evolution (Arnab et al., 2025a). The extracted features were then used to train a smaller task-specific classifier to discriminate between selective sweeps and neutrality. This approach, termed transfer learning (Bozinovski, 2020), eliminates the need to train a large network from scratch, thus reducing computational and energy costs, minimising the demand for extensive data and producing feature representations that are more robust to noise. Importantly, this framework performs at least as well as other methods for detecting natural selection from image encodings of genome alignments, demonstrating how advances in feature generation and extraction can drive significant progress in evolutionary inference.

**Figure 2:**
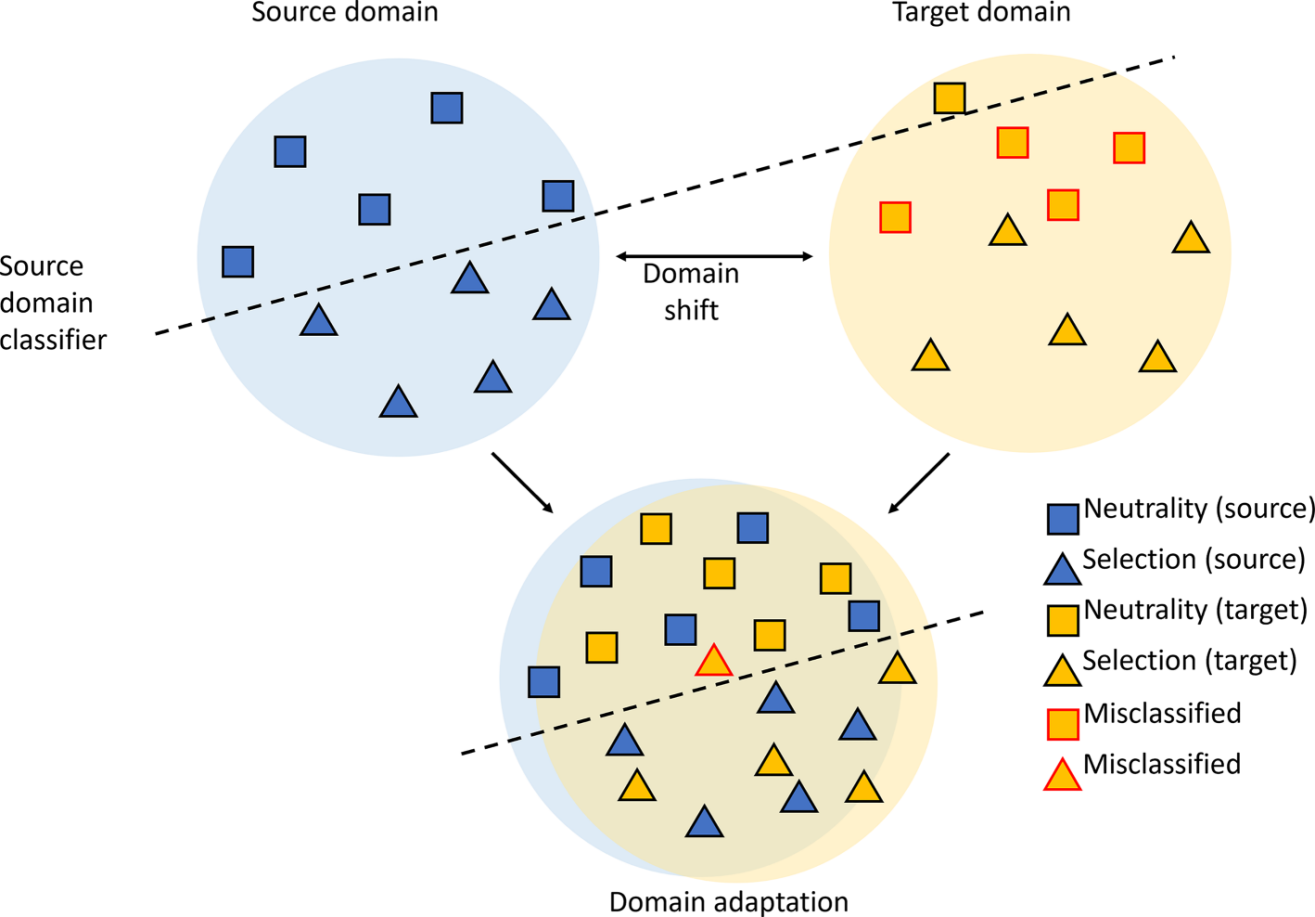
Domain adaptation to circumvent domain shift when inferring evolutionary events. Source and target domains fall into distinct regions of input space due to domain shift, which results in a classifier trained on the source domain potentially misclassifying observations from the target domain. Application of domain adaptation to obtain a common domain between the source and target results in minimal misclassification.

A form of transfer learning that is particularly important for studying nonmodel organisms is domain adaptation (Figure 2). Specifically, synthetic samples are typically simulated to generate training data (termed the source) to build machine learning models for downstream prediction tasks on empirical data (termed the target). However, generating these training samples requires an array of assumptions, including the distribution of genetic parameters (e.g., mutation and recombination rates), demographic history, and adaptive processes, many or all of which may be unknown or poorly estimated in nonmodel systems. Moreover, synthetic data are typically error-free unless noise is explicitly injected, whereas data from nonmodel systems are often fraught with uncertainty due to low-coverage genomes or the lack of resources to generate high-quality variant calls, as previously suggested. Therefore, the distributions (domains) of source and target data are typically misaligned, leading to a phenomenon called domain shift (Figure 2), which can produce spurious inferences when machine learning models trained on synthetic source data are applied to empirical targets.

**Figure 3:**
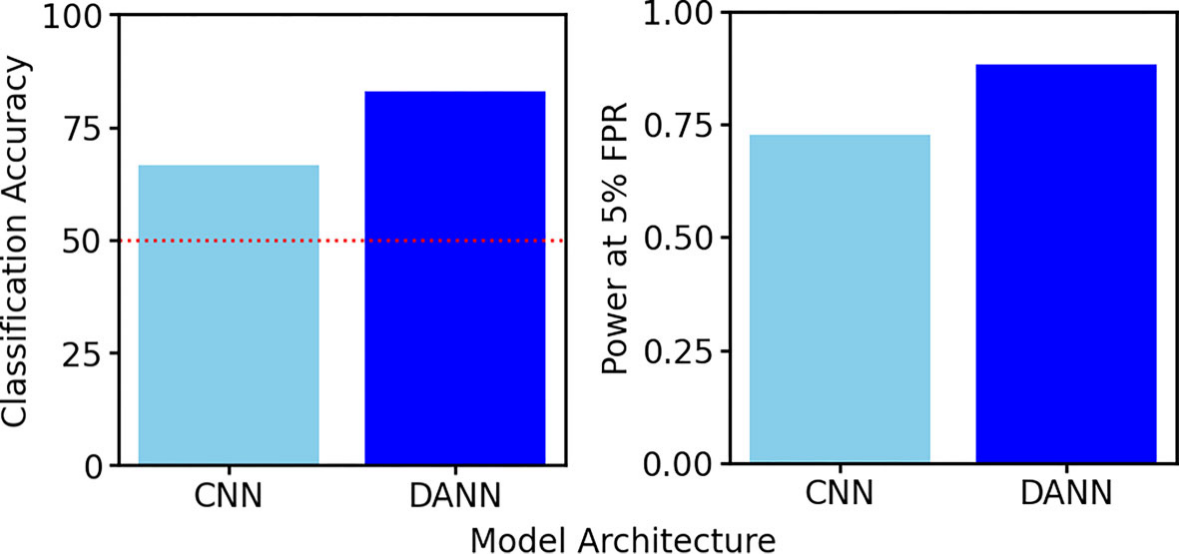
Classification accuracy and power at 5% false positive rate (FPR) for both a standard CNN and a DANN applied to image representations of multilocus genotype variation (Figure 1). The setting is difficult, as it represents models trained and tested on multilocus genotype data from 10 sampled diploid individuals. Each model is trained on known variation under a constant population demographic history, whereas it is tested on a population bottleneck history in which variation is estimated based on expected minor allele counts (see Figure 1). The DANN model is better able at detecting sweeps under domain shift than the standard CNN under this challenging setting with small sample size, unphased genotype variation, and demographic and genotype uncertainty.

Given the limited knowledge about the target domain in nonmodel systems, unsupervised domain adaptation is usually applied, where each source sample has both input features and an output label, whereas the target samples are unlabelled and have only input features. To date, such methods have been applied to detect adaptive processes and estimate recombination rates and selection coefficients (Mo & Siepel, 2023; Song et al., 2024). These studies have primarily used Domain Adversarial Neural Networks (DANNs; Figure 3) (Ganin et al., 2016), which apply adversarial learning between two competing players: a label predictor that infers output labels (e.g., neutrality or selection) and a domain discriminator that classifies whether a sample originates from the source or target domain. Both models operate on feature representations generated by a deep neural network, termed the feature generator, which processes the input features.

The goal of DANNs is to learn robust feature representations that are discriminative simultaneously for the task of predicting the output label and invariant to the domain. This objective is achieved by minimising the errors in label predictions while maximising the error of the domain classifier, thus aligning the source and target domains in the feature space while preserving the relevant information for the task. DANNs accommodate “global” distribution shifts by aligning the marginal distributions of source and target feature representations such that these distributions are similar after training. This alignment makes them particularly useful when the domain shift is modest or expected to be uniform across labels, such as shifts induced by coverage variation, low mappability, or alignment uncertainty. However, more sophisticated approaches are needed when the source and target distributions differ more substantially or in class-specific ways, such as different shifts for neutral regions and those under selection. Because DANNs ignore class information when aligning the domains, they may fail to capture finer-scale differences between classes. We outline potential future avenues for tackling such complex domain shifts. Improvements can arise by accounting for “local” shifts between class-conditional distributions, where models align the probability distribution of the feature representations conditional on the class label for the source and target.

Multi-Adversarial Domain Adaptation (MADA) (Pei et al., 2018) tackles this limitation by refining how the model aligns the source and target domains. Instead of using a single domain discriminator, which is a network whose sole purpose is to tell whether a sample came from the source or the target, MADA introduces multiple discriminators, with one for each class of interest (e.g., neutrality or selective sweep). Each class-specific discriminator attempts to align only the subset of feature representations associated with its corresponding class.

By aligning each class separately, the model can better preserve class-specific structure in the data. This approach encourages positive transfer, meaning that information from source samples belonging to a given class is correctly transferred to target samples from the same class. At the same time, it reduces negative transfer, which occurs when information from one class in the source (e.g., neutrality) incorrectly influences target samples belonging to a different class (e.g., selective sweep). In doing so, MADA permits the model to handle situations in which domain shift differs across classes, such as when selective sweeps and neutral regions are affected differently by demographic history or data quality. MADA is particularly useful when only a small fraction of the genome may show adaptive signals, while the majority behaves neutrally, as is expected for many organisms. In such cases, global aligners like DANN may be misled by the dominance of the neutral signal, whereas MADA can enable localised natural selection-aware alignment.

DANNs and MADA can be viewed as two ends of a spectrum of domain alignment strategies. Dynamic Adversarial Adaptation Networks (DAANs) (Yu et al., 2019) introduce a tunable parameter to interpolate between them. This flexibility enables DAANs to model complex domain shifts that result from interactions of demographic and selective processes, such as the influence of bottlenecks on genomic variation that may confound selection inference. Importantly, the additional parameter is implicitly estimated during training, ensuring that DAANs maintain computational efficiency despite their expressiveness.

One challenge with DAANs, inherited from MADA, is the need to train multiple domain discriminators, which can be computationally intensive. An alternative strategy is to align the joint distributions of feature representations and labels, rather than the marginal or class-conditional ones. Conditional Domain Adversarial Networks (CDANs) (Long et al., 2018) adopt this approach by modelling the joint representation of learnt features and class label. CDANs aim to align these joint distributions for source and target using a single discriminator, capturing the dependencies between feature representations and classes. While this approach introduces more parameters from a new higher-dimensional joint input, it allows to better capture how demographic and adaptative processes jointly shape genomic patterns.

Recent ensemble-based approaches may offer further advantages in settings with noisy or uncertain target data, such as those involving low-coverage sequencing or ancient DNA, by improving robustness during domain alignment. One such method is Co-regularised Adaptive Alignment (Co-DA) (Kumar et al., 2018), which still uses an adversarial domain discriminator, but introduces two label predictors that are penalised when their predictions disagree on the target. This co-regularisation encourages stability and agreement in low-confidence regions. In contrast, Adversarial Dropout Regularization (ADR) (Saito et al., 2018a) and Maximum Classifier Discrepancy (MCD) (Saito et al., 2018b) do not employ domain discriminators. ADR instead uses dropout perturbation to simulate uncertainty in the target feature representations, encouraging the boundary between classes to fall in low-density regions of feature space. MCD also uses two label predictors, but trains them to maximise their disagreement on the target for a fixed feature generator, and then updates the generator to minimise this discrepancy. This two-stage training encourages clear separation between classes and effective class-conditional alignment. Like CDANs, these methods attempt to align joint feature-label distributions between the source and target, making them particularly suitable alternatives to CDANs for noisy settings.

In cases where geometric differences between source and target domains are substantial, such as from differences in recombination landscapes, or due to sampling from spatially- or temporally-structured populations, explicitly aligning the geometric structure of the joint distributions may be necessary. Joint Distribution Optimal Transport (JDOT) (Courty et al., 2017) provides such a solution by aligning the joint feature-label distributions of the source and the target using an optimal transport formulation, which identifies the most efficient approach to transform one distribution into another. Unlike adversarial methods, JDOT directly minimises an objective function that combines discrepancies in both the generated feature and the label space, leading to more stable alignments. JDOT is also well-suited to regression problems, enabling continuous inference of recombination rates or selection coefficients across domains.

Although not originally developed to address domain shift, recent work has shown that it is possible to detect specific adaptive signals without explicitly modelling the distribution of other processes (Arnab et al., 2025b). This approach employs positive unlabelled learning (Elkan & Noto, 2008), where the user provides training data representing the target positive class (e.g. selective sweeps or another adaptive process), but is not required to provide examples of the complementary negative class (e.g., neutrality). Instead, the method treats the unlabelled data as a mixture of positive and negative cases and iteratively learns to distinguish between them. In practice, this means genomes are classified as either consistent with the target class (e.g., sweep) or inconsistent with it (e.g., non-sweep), with the model inferring what non-sweep looks like directly from the empirical genomic distribution to which it is applied. This positive unlabelled learning framework has been shown to effectively detect sweeps without prior knowledge of the underlying demographic or genetic parameters (Arnab et al., 2025b), which makes it particularly well suited for applications in nonmodel systems, where such information is often limited or uncertain.

Finally, contemporary advances in AI have entered population genetics through transformer- and language-model approaches (Radford & Narasimhan, 2018) for representing genomic variation. A recent study (Korfmann et al., 2025) introduced a model that learns coalescence-time distributions conditional on mutational context from diverse simulated data and infers ARGs autoregressively across chromosomes. These inferred coalescence times and recombination patterns can be used to study evolutionary forces, with unusually recent or ancient times suggesting positive or balancing selection, respectively (Hejase et al., 2020). The model matches state-of-the-art performance for coalescence-time and demographic inference, its empirical predictions align with known adaptive regions, and remains robust under domain shift (Figure 2), enabling accurate inference across species. Together, these properties position it as the first general-purpose pre-trained (foundation) model for evolutionary analysis in both model and nonmodel systems.

## Explainable and interpretable models

In addition to all technological advances discussed so far, one critical step for AI to be widely used in evolutionary genomics of nonmodel species is to be explainable, interpretable, accessible, and inclusive. Once labelled a *black-box* model, AI is now becoming increasingly more transparent thanks to the introduction of techniques to “explain” and “interpret” its predictions, which should be fair, robust, and trustworthy. Explainability is model-centric and it is a prerequisite for interpretability, which is human-centric. The motivation beyond explainable AI models is to make their predictions and behaviour, when relevant, more understandable by users (Gunning et al., 2019). Although explainable AI has a fast-paced and dynamic community (Speith, 2022), achieving it is difficult for several reasons, including that models are complex, users vary in expectations and expertise, and explanations can span a wide spectrum of possibilities.

Explanations may not always be necessary for some AI applications in evolutionary genomics, where synthetic training data are often generated from well-understood models. Additionally, one may argue that there is less potential harm in having opaque models in evolutionary genetics than, for instance, clinical genetics. Finally, evolutionary genomics practitioners may be less interested and less qualified in understanding the inner mechanisms of deep neural networks. This is an important notion, as explainable AI (and its assessment) depends on the task and the technical skills, experience, and expectations of the user. Therefore, users in this field may favour highly performant models over explainable ones, as there is a general trade-off between these two qualities. For example, generative and large-language models, which are becoming increasingly popular in the field (Korfmann et al., 2025; Yelmen & Jay, 2023), have notoriously poor explainability due to their large number of parameters. However, a move toward building interpretable AI models is particularly needed for the study of nonmodel species, where the lack of extensive prior studies or theoretical foundations hampers any *a posteriori* justification of its predictions. Practitioners could be interested in understanding, for instance, which part of their data has contributed to a particular prediction to assess the robustness of their dataset or to test their original hypotheses or generate novel insights. Without the support of tools to explain AI computations, researchers can easily not realise fallacies in its predictions and come to wrong biological conclusions, for example due to poor model capacity and its inability to predict certain classes.

In evolutionary genomics, AI models tend to be partially interpretable, which means that they can explain finite components of their computing, rather than giving the full picture. In this approach, a model can provide *post hoc* explanations of its features on a simplified model after training. This is in contrast to *ante hoc* explainable models, such as decision trees, which are interpretable by design. Commonly used importance metrics to highlight which measures were most influential in a prediction include SHAP (Lundberg & Lee, 2017) and LIME (Ribeiro et al., 2016) values. These approaches provide local explainability as they generate some understanding of aspects for single predictions. They have been used successfully in the field, for example, to identify the most important landscape and environmental characteristics that explain the geographical distribution of genetic diversity (Kittlein et al., 2022) or in demographic inferences (Quelin et al., 2025). However, *post hoc* can sometimes be fragile to small perturbations at the input.

**Figure 4:**
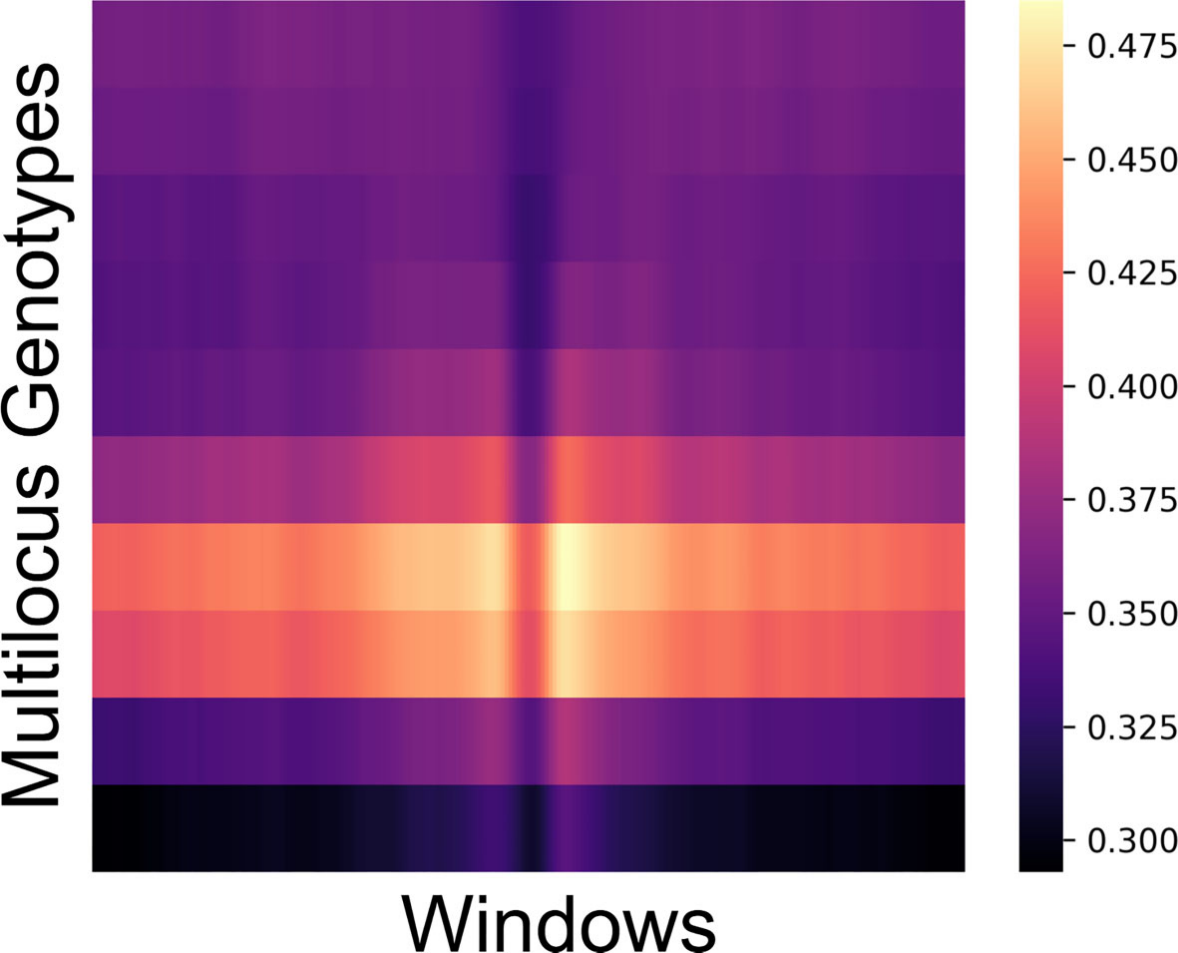
Heatmaps summarizing mean GradCAM maps from a DANN model (Figure 3) applied to training data, with the mean taken across 2,000 training observations with 1,000 observations from each of the neutral and sweep classes. The heatmap highlights the regions where the model focuses most strongly when distinguishing selective sweeps from neutrality. Most of the emphasis is concentrated around the seventh and eighth multilocus genotypes from the top, near the boundary of the dark region centered on the locus of selection (see Figure 1 as reference), suggesting that the model relies heavily on local intensity transitions around the center pixels to identify sweeps. The attention gradually fades around the fifth and sixth as well as the ninth and 10th multilocus genotypes from the top, likely reflecting redundancy in the surrounding signal where neighboring genotypes convey similar information.

Another attempt to provide local explanations is by using saliency maps (Simonyan et al., 2014) or gradient-based methods such as Grad-CAM (Selvaraju et al., 2017) (Figure 4), which are popular methods in computer vision to highlight the most informative parts of an image. In evolutionary genomics, informative pixels (e.g., alleles or genotypes) of aligned genomes for a specific task (e.g., detection of selective sweeps) can provide insights into the data patterns used by the model (e.g., linked sites in hitch-hiking) (Gower et al., 2021). Narratives can then be built to relate individual alleles or genotypes to broader concepts or patterns from experience or intuitions. However, heatmaps that summarise the relative importance of each pixel can be hard to interpret for complex tasks where there are no clear expectations. In this scenario, building a narrative is problematic and users could be unconsciously biased to interpret patterns as a validation of their original hypotheses. Finally, deciphering the inner layers or activations of neural networks provides global explanations of how the AI model works. These approaches have not yet been fully explored in evolutionary genomics.

Beyond existing strategies based on attribution methods, novel frameworks that favour feature extraction and selection are emerging in genomics. Some of them are particularly suitable for temporal (e.g., trajectories of allele frequencies) or tabular data (e.g., genotype-phenotype associations). In this spirit, biologically inspired features have been proposed as a way to achieve high-performance *post hoc* explainable models in genomics (Hequet et al., 2025). Interpretable neural networks for evolutionary inferences have also been devised by correlating known summary statistics with the values of the hidden (and last) layers (Riley et al., 2024). Furthermore, interpretability has been achieved by permuting input data (e.g., haplotype alignments) and assessing the loss in predictive performance (Tran et al., 2025). These analyses revealed how a model-agnostic network recapitulated existing summary statistics and weighted their importance in prediction. Finally, reliability scores have been developed to assess the trustworthiness of detecting outliers from a supervised machine learning model (Ahlquist et al., 2023). This approach allows for the link between AI predictions with theoretical models and empirical observations. A potential new direction for explainable AI models in evolutionary genomics would be to embrace recent advances in bridging the gap between local and global explainable AI (Achtibat et al., 2023). Here, we could consider individual pixel predictions as semantically rich concepts, such as known (but ideally new) summary statistics. By concurrently identifying summary statistics learnt by the model and relating them to each input data, we could potentially generate user-centric explanations.

It is difficult to assess which explainable methods are the most suitable, as different users may have a different understanding of the explanations provided. However, here we argue that, when building new AI models in evolutionary genomics, a shift toward user-centred explainable but still highly performant models should be preferred. This ambitious goal can be achieved through cross-domain collaborations, with practitioners lending their system expertise to modellers (Longo et al., 2024). Ultimately, AI inferences should be accompanied by a collection of methods for model explanability to reach a consensus on the most likely explanations for a given task or model (Seuß, 2021). Robust interpretations naturally emerge by aggregating single explanations and focus on a human-centred approach to transparency. More transparent and interpretable models will further accelerate the adoption of AI models for the study of nonmodel species.

## Accessibility and inclusion

In addition to being explainable and interpretable, AI models for the study of nonmodel species should be accessible and inclusive. However, access to dedicated local computing resources or to expensive cloud solutions is still a barrier to poorly funded research groups. In addition to the computational cost of training neural networks, a major bottleneck in evolutionary genomic inferences using AI is the need to generate extensive simulations to create a training dataset. As rescaling parameters to run efficient simulations can bias some features of the data (Dabi & Schrider, 2025; Marsh et al., 2025), it is imperative to consider solutions that rely on fewer simulations. Sharing pre-trained networks for transfer learning, for instance, from extensive simulations on a wide collection of species models (Korfmann et al., 2025; Lauterbur et al., 2023a), would allow users to only focus on generating few but targeted simulations for fine-training. The hardware accelerator designs for CNNs have been successfully tested to detect selective sweeps and have shown a dramatic increase in performance (Alachiotis & Souilljee, 2025). In addition to user-friendly implementations (Zhao et al., 2023), such improvements contribute to making genome-wide scans more cost-effective than they currently are. All of these considerations will also reduce the environmental cost of training AI algorithms.

Scientists can find a wide collection of resources to train themselves in using the latest AI technologies. However, few resources are dedicated to evolutionary applications. This is a potential issue given the conceptual differences in employing AI in evolutionary genomics (e.g., synthetic training data). Although scientists often deposit their code and trained networks as part of their disseminations, new users are required to make significant efforts to adapt implementations to their particular task and system. In other words, making an implementation transparent does not necessarily imply its inclusion. Rethinking how we design new algorithms by focussing on inclusion first and then transparency will bring a fresh new interpretation of open science beyond the mere sharing of data and resources (Leonelli, 2023). We also urge novel practitioners, especially early career researchers, to follow best-practice guidelines on the use of AI (Crilly et al., 2025) to avoid falling into the pitfall of brute-force using AI without clear needs or hypotheses.

## Conclusions

The rapid adoption of AI in evolutionary genomics is a black swan event: unpredictable with major consequences but then rationalised in hindsight. From a long history of theoretical and empirical work, a move toward a data-driven approach in this field is inevitable and welcomed. This revolution is now possible thanks to the fast-paced development and usage of deep learning algorithms in evolutionary genomics, now finally accessible to the study of nonmodel species. In addition, AI models are particularly suitable for embedding metadata or diverse sources of information, making them particularly appealing for applications linking genetic data with environmental factors, morphometric measurements, or phenotypes.

Here, we emphasised how AI can democratise access to advanced analysis and can remove barriers traditionally imposed to researchers studying nonmodel species. We argue that a new paradigm shift is in dire need in the methodological advancement of evolutionary genomics. We should exploit the supremacy of deep learning and other AI approaches to develop tools that are sufficiently general to be applicable to a wide variety of systems and datasets, and consider their application to model species as a mere case study. In this framework, we could reverse the trend of diminishing disruptive science, which is generally observed in different disciplines (Park et al., 2023). However, caution and good practice in the responsible use of AI should be paramount.

Evolutionary genomic studies of nonmodel species represent an opportunity rather than a limiting factor in using AI, as novel challenges motivate new technological solutions that can be more generalisable to other systems. In this scenario, empiricists will be the driving force behind establishing AI as a powerful and accessible inferential tool in evolutionary genomics of nonmodel species.

## Supplementary Material

qrag004_Supplemental_File

## Data Availability

The open-source implementation is available at https://github.com/sandipanpaul06/DANN, and the simulated replicates through Zenodo at https://doi.org/10.5281/zenodo.18166412.
