# Supplementary material for: AI solutions for evolutionary genomics of nonmodel species
Source: Evol Lett. 2026 Mar 3;10(2):135–46. doi: 10.1093/evlett/qrag004 (PMC13043892; doi:10.1093/evlett/qrag004)
Supplement: qrag004_Supplemental_File [file qrag004_supplemental_file.pdf]

Supplementary Material

AI solutions for evolutionary genomics of nonmodel species

Michael DeGiorgio[1,2,3], Sandipan Paul Arnab[1,3] Matteo Fumagalli[4,5]

[1]Department of Electrical Engineering and Computer Science, Florida Atlantic University, Boca Raton, FL, USA
[2]Department of Biomedical Engineering, Florida Atlantic University, Boca Raton, FL, USA
[3]Center for Omics technologies and Data Engineering, Florida Atlantic University, Boca Raton, FL, USA
[4]School of Biological and Behavioural Sciences, Queen Mary University of London, Mile End Road, E1 4NS, London, United Kingdom
[5]The Alan Turing Institute, 96 Euston Road, NW1 2DB, London, United Kingdom

AI implementation to detect selective sweeps

We employed the coalescent simulator `discoal` to generate both neutral and sweep replicates under two demographic models: a bottleneck demographic history and a constant-sized demographic history. In the bottleneck model, the population size is 15,000 diploids at present, contracts to 5,000 diploids between 1,500 and 2,000 generations ago, and returns to an ancestral size of 10,000 diploids prior to 2,000 generations. This demographic history produces a time-averaged diploid effective population size (N_e) of approximately 10,000 diploids, based on cumulative coalescent intensity, ensuring comparability with the constant-size scenario. To simulate selective sweeps for both models, we drew per–generation selection coefficients (s) uniformly at random from the interval $[0.01, 0.1]$, set initial beneficial allele frequencies (f) by sampling uniformly at random on a logarithmic scale from $[1/(2N_e), 0.1]$, and chose fixation times (τ) uniformly at random within $[0, 1000]$ generations prior to sampling. Per-site per-generation mutation rate was set to $\mu = 1.25 \times 10^{-8}$, and the per-site per-generation recombination rate was set to $r = 10^{-8}$. For each simulated replicate, we sampled 20 haplotypes of length 1.1 megabases.

We implemented an illustrative algorithm to detect selective sweeps with genotype and demographic uncertainty. The open-source implementation is available at https://github.com/sandipanpaul06/DANN. We have also released the simulated replicates through Zenodo (https://doi.org/10.5281/zenodo.18166412). The algorithm is based on a DANN architecture consisting of three main components: a feature extractor, a class predictor, and a domain classifier connected through a gradient reversal layer (GRL). The feature extractor employs two convolutional blocks, each containing a convolutional layer with kernel size of 3×3 and 32 filters, batch normalisation, rectified linear unit (ReLU) activation, and 2×2 max pooling with stride two, followed by a flattening operation and a fully connected layer with 128 units. This shared feature extractor learns representations from both source and target domain samples. The label predictor is a single-node output layer with sigmoid activation that performs binary classification on the extracted features, trained exclusively on labelled source domain samples using binary cross-entropy loss. The domain classifier, which aims to distinguish between source and target domains, consists of a two-node softmax output layer trained with categorical cross-entropy loss that receives input through a GRL. During forward propagation, the GRL acts as an identity function, but during backpropagation, it multiplies gradients by

-1 before passing them to the feature extractor.

To train the DANN model under the domain-mismatch scenario, we assembled a training set consisting of 1,000 sweep and 1,000 neutral replicates from both the constant population size and bottleneck demographic histories. Replicates from the constant population size history were provided with both class labels (sweep or neutral) and a domain label of zero. Replicates from the bottleneck history were assigned only a domain label of one, while their class labels were masked during training and excluded from the classification loss calculation. The validation and test sets were constructed identically, each containing 1,000 sweep and 1,000 neutral replicates from both demographic histories.

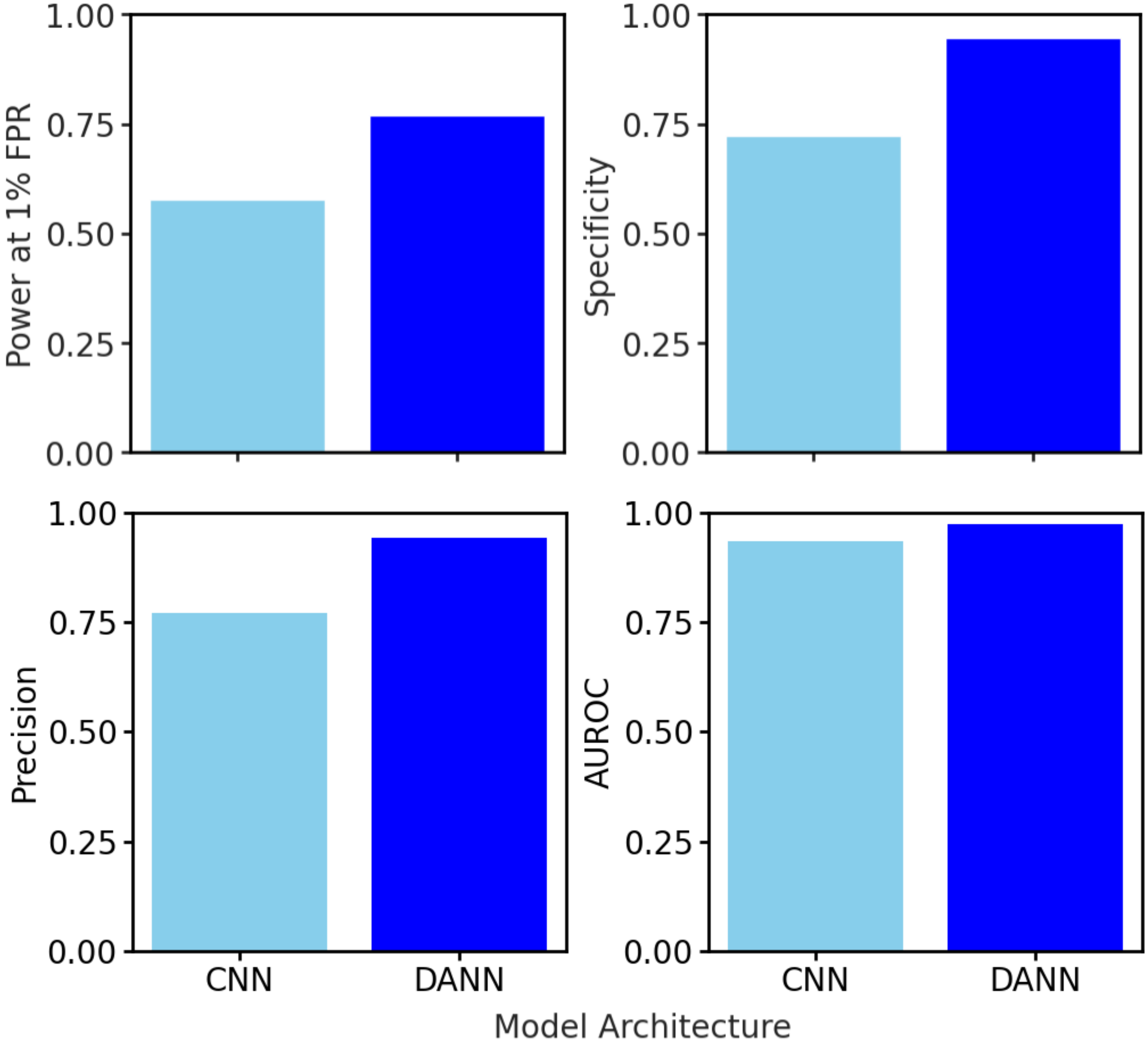

Figure S1: Power at a 1% false positive rate (FPR), specificity, precision, and area under the receiver operating characteristics curves (AUROC) for both a standard CNN and a DANN applied to image representations of multilocus genotype variation. The setting is difficult, as it represents models trained and tested on multilocus genotype data from 10 sampled diploid individuals. Each model is trained on known variation under a constant population demographic history, whereas it is tested on a population bottleneck history in which variation is estimated based on expected minor allele counts. The reported metrics quantify the ability of a classifier to correctly detect true selective sweeps, minimize misclassification of neutral regions, and maintain robust discrimination performance across decision thresholds. Across all four metrics, DANN comfortably outperformed a standard CNN.
